# A one-week reduced-carbohydrate diet lowers insulin requirements and shifts the IGF axis with no detectable short-term change in endothelial function in a randomized, crossover trial of adults with type 1 diabetes

**DOI:** 10.1186/s40842-026-00276-6

**Published:** 2026-03-26

**Authors:** M. Naweed Akbar, T. Jordan Smith, Sivaprakasam Chinnarasu, Wendi Welch, Wang Zuofei, Bridget Litts, Lauren M. LeStourgeon, Mohammad Saleem, Mohd Mabood Khan, Annet Kirabo, Justin M. Gregory

**Affiliations:** 1 Ian Burr Division of Pediatric Endocrinology and Diabetes, Vanderbilt University School of Medicine, 1500 21st Avenue South, Suite 1514, Nashville, TN 37212-3157 USA; 2 Division of Diabetes, Endocrinology and Metabolism, 2213 Garland Avenue/ MRB 4, Nashville, TN 37232 USA; 3 Department of Internal Medicine, Vanderbilt University School of Medicine, 1161 21st Ave S, Nashville, TN 37232 USA; 4 Division of Genetic Medicine and Clinical Pharmacology, 2220 Pierce Avenue, 560 Preston Research Building, Nashville, TN 37232 USA

**Keywords:** Type 1 diabetes, Reduced‑carbohydrate diet, Insulin dose, GH–IGF‑1 axis, IGFBP‑1, IGFBP‑3, Flow‑mediated dilation, Endothelial function

## Abstract

**Background:**

Individuals with type 1 diabetes mellitus (T1DM) must deliver insulin into the peripheral circulation rather than more physiologically into the hepatic portal circulation, leading to a chronic state of underinsulinization in the liver. Previous research suggests that decreased hepatic insulin perturbs the growth hormone-IGF-1 system in these patients. We tested whether short-term carbohydrate restriction, by lowering exogenous insulin requirements and exacerbating hepatic underinsulinization, modifies IGF axis hormones, binding proteins, and vascular function.

**Methods:**

We performed a secondary analysis of plasma samples collected during a single-blind crossover study of twelve adults with T1DM using automated insulin delivery. In random order, the participants consumed a one-week reduced carbohydrate diet (RCD) and an isocaloric standard carbohydrate diet (SCD), each followed by a study visit. We measured total and free IGF-1 and IGF-binding proteins (IGFBP-1, IGFBP-2, and IGFBP-3) after overnight fasting and during the final 30 min of a hyperinsulinemic-euglycemic clamp. We also measured endothelial function using brachial artery flow-mediated dilation (FMD).

**Results:**

The RCD lowered the total daily insulin dose versus SCD (16% during the week and 24% in the 24 h before testing), with similar glucose levels across diets. Compared with SCD, RCD reduced total IGF-1 and IGFBP-3 and increased IGFBP-1 and IGFBP-2 at baseline. These patterns also persisted during insulin-stimulated conditions. Free IGF-1 was more variable and did not differ significantly between diets. Despite clear shifts in the IGF axis, FMD did not differ between diets and did not correlate with IGF axis markers after either intervention.

**Conclusions:**

In adults with T1DM studied under nearly matched glycemia, one week of carbohydrate reduction lowered insulin requirements and shifted the IGF axis in a pattern akin to reduced portal insulin exposure, without detectable changes in conduit artery endothelial function. These findings identify the IGF axis as a sensitive physiologic readout of insulin exposure. Longer-term studies are needed to determine whether sustained changes in the IGF axis impact vascular biology or cardiometabolic risk.

**Trial registration:**

ClinicalTrials.gov NCT04118374.

**Supplementary Information:**

The online version contains supplementary material available at 10.1186/s40842-026-00276-6.

## Research insights

**What is currently known about this topic?** • Peripheral insulin delivery underinsulinizes the liver in type 1 diabetes (T1DM). • Decreased liver insulin alters GH-IGF-1 signaling. • IGF axis abnormalities may contribute to vascular risk in T1DM.

**What is the key research question?** • Does lowering insulin using a reduced carb diet modify the IGF axis and vascular risk in T1DM?

**What is new?** • A one-week reduced carb diet reduced insulin doses by 16% without worsening glycemia.• After the reduced carb diet, IGF-1 and IGFBP-3 decreased; IGFBP-1 and IGFBP-2 increased. • Endothelial function was similar between reduced and standard carb diets in this crossover study.

**How might this study influence clinical practice?** • Diet-driven insulin reduction shifts the IGF axis; clinical impact needs further characterization.

**Supplementary Information:**

The online version contains supplementary material available at 10.1186/s40842-026-00276-6.

## Graphical Abstract

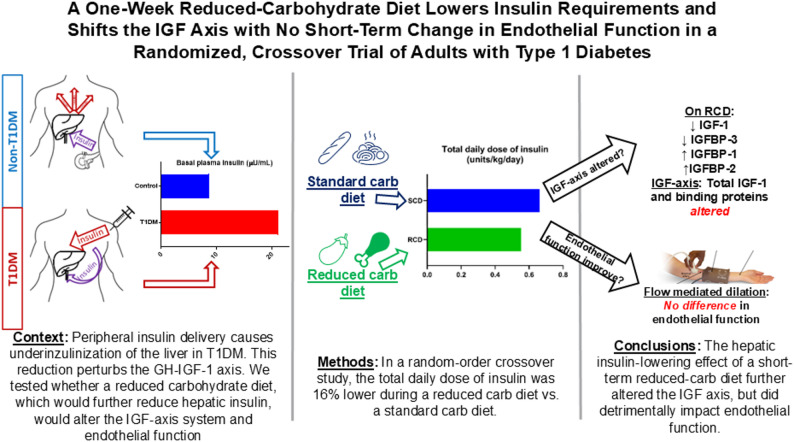

## Introduction

People with type 1 diabetes mellitus (T1DM) experience metabolic disturbances beyond chronic hyperglycemia, contributing to persistently elevated cardiovascular disease risk even when achieving glycemic targets [1]. Among the least-appreciated disturbances are abnormalities of the insulin-like growth factor (IGF) system. In multiple T1DM cohorts, investigators have found subnormal circulating IGF‑1 concentrations, while its insulin‑sensitive binding protein, IGFBP‑1, is elevated [2, 3]. This pattern reflects hepatic resistance to growth hormone (GH), driven by inadequate insulin signaling in the portal circulation. Low portal insulin impairs hepatic IGF-1 synthesis, which in turn reduces feedback inhibition of pituitary GH secretion. Simultaneously, low portal insulin exposure directly stimulates hepatic IGFBP-1 production [2, 4, 5]. IGFBP-3 is the primary carrier of IGF-1 and is often decreased, especially in individuals with chronic diabetic complications [6–8]. Collectively, these changes reduce IGF-1 bioactivity, diminishing the hormone’s protective effects on vascular health and potentially linking hepatic insulin deficiency to increased vascular disease risk in T1DM [3].

Because insufficient hepatic insulin drives these IGF axis abnormalities, restoring insulin delivery to the portal circulation can substantially rectify them. For example, in adults with T1DM, intraportal insulin infusion significantly increases hepatic IGF-1 production, restoring negative feedback on pituitary GH secretion thereby lowering GH concentrations [9–11]. Portal insulin also suppresses IGFBP-1 production, normalizing its circulating levels. These beneficial effects on the IGF-1 axis are not fully achievable with conventional subcutaneous insulin therapy [9–11]. Peripheral insulin delivery bypasses the liver, leaving it relatively insulin-deprived and sustaining low IGF-1 alongside elevated IGFBP-1 and GH levels [5, 11]. These disturbances represent more than biochemical curiosities, as they may actively contribute to the long-term complications associated with T1DM. Studies in T1DM and T2DM have linked reduced IGF-1 activity to microvascular dysfunction and accelerated vascular aging [8, 12]. Elevated IGFBP-1 and IGFBP-2 may further aggravate vascular risk by sequestering IGF-1, diminishing its bioavailability and protective vascular effects [3, 13]. Together, these observations underscore the insulin-GH-IGF axis as a mediator linking portal insulinization with metabolic and vascular health in T1DM [14].

Even when intensive insulin therapy achieves glycemic goals, cardiovascular risk remains elevated in T1DM, suggesting that factors beyond hyperglycemia per se drive vascular injury [8, 15]. Several groups, including ours, have investigated the cardiometabolic consequences of chronic iatrogenic hyperinsulinemia that results from peripheral insulin delivery. Injecting insulin into peripheral tissue, rather than pancreatic insulin draining into the portal vein, leads to systemic hyperinsulinemia and relative hepatic insulin deficiency [16]. Our previous research in canines and humans implicated this imbalance as a contributor to insulin resistance and glycemic variability in T1DM [17–20]. This altered peripheral-to-portal insulin gradient may also perpetuate hepatic GH resistance, reduce IGF-1 production, and elevate IGFBP-1 and IGFBP-2, further amplifying vascular risk [9–11, 21]. Thus, restoring a more physiological portal-to-peripheral insulin balance while maintaining glycemic control might improve both the IGF axis and associated metabolic and vascular physiology in T1DM [3, 13, 14].

Recently, we demonstrated in a randomized crossover study that a one-week reduced-carbohydrate diet (RCD) significantly reduced peripheral insulin requirements but did not improve insulin sensitivity or endothelial function compared with a standard-carbohydrate diet (SCD) [22]. However, that trial did not assess IGF-1 or its binding proteins, biomarkers that reflect hepatic insulinization and that have been associated with microvascular complications and adverse cardiovascular outcomes in observational studies [8, 12, 23–26]. Dietary manipulation is a practical way to test how altering peripheral insulin exposure influences the portal-to-peripheral insulin gradient [25, 27]. Reducing carbohydrate intake lowers systemic insulin requirements and peripheral hyperinsulinemia but concurrently intensifies relative hepatic insulin deficiency. Still, it remains unclear whether a clinically achievable reduction in insulin requirements—without changing the route of insulin delivery and without worsening glycemia—is sufficient to shift circulating IGF axis biomarkers in T1DM. Defining the magnitude and direction of these responses may help interpret the cardiometabolic trade-offs of insulin-sparing strategies and inform future approaches aimed at restoring hepatic insulinization.

Here, we extend our recent work by testing whether altering peripheral insulin exposure—and consequently altering the hepatic insulin gradient—impacts the IGF-1/IGFBP axis and vascular endothelial function. We hypothesized that the RCD, by further reducing hepatic insulin exposure, would shift the IGF axis toward a profile resembling portal insulin deficiency, characterized by lower IGF-1 and increased insulin-sensitive IGFBPs [11]. In contrast, we expected an isocaloric SCD, requiring higher peripheral insulin doses, would partly restore hepatic insulin exposure and thus maintain higher IGF-1 levels [2, 22]. We also examined whether these insulin-dependent hormonal changes were accompanied by differences in endothelial function, as measured by flow-mediated dilation (FMD) [13, 22].

## Methods

### Participants

We performed a secondary analysis of plasma samples collected during a previously reported randomized crossover trial [NCT04118374; [22]. Adults aged 18–60 years with T1DM, a BMI of 18–33 kg/m^2^, and HbA1c of 5.9-9.0% were eligible. Exclusion criteria included severe hypoglycemia within the past three months, diabetic ketoacidosis within past six months, current pregnancy, anemia, and use of medication affecting insulin sensitivity or endothelial function. (see Supplemental Table 1 for complete criteria). Participants were recruited from the Vanderbilt Eskind Diabetes Clinic and community resources. Enrollment occurred between November 2021 and March 2024.

### Study design

We conducted a single-blind, random-order crossover study to compare the effects of an RCD versus an SCD on IGF-related hormonal outcomes. The crossover design allowed participants to serve as their own control, reducing variability and increasing statistical power. After an initial screening visit where baseline characteristics were obtained prior to randomization, participants underwent two separate one-week dietary intervention periods, each followed by inpatient research visits at Vanderbilt’s Clinical Research Center, as detailed previously [22].

The two one-week diet periods were separated by a three-week washout to minimize carryover and to facilitate scheduling study visits during the follicular phase of the menstrual cycle for female participants. This interval substantially exceeds the time course over which insulin-linked IGF-axis components change. For example, circulating IGF-I is predominantly carried in IGFBP-3-ALS ternary complexes with a half-life on the order of 12–15 h, and IGFBP-1 responds to changes in insulin exposure over hours [28, 29].

### Diet intervention

Participants received two tailored, isocaloric one-week diet plans using the Mifflin-St. Jeor formula adjusted for activity levels. The SCD provided 45–65% of energy from carbohydrates, 10–30% from protein, and 25–35% from fat. The RCD reduced carbohydrate intake to 25–35%, increased fat intake to 45–65%, and maintained protein intake at 10–30%. This macronutrient adjustment aimed to decrease the total daily insulin requirement substantially—approximately 25% lower compared to SCD—while avoiding the extremes of very low-carbohydrate diets. During the 24 h prior to each inpatient visit, participants consumed study-provided, pre-portioned 2,000-kcal meals whose macronutrient composition matched the assigned diet (RCD vs. SCD). Participants recorded dietary adherence via detailed food logs (MyFitnessPal), reviewed by study staff. For macronutrient analyses, we summarized intake using participants with complete logs for all seven days of each intervention.

### Research visit

Participants completed standardized inpatient assessments of vascular function and insulin sensitivity, including FMD and a hyperinsulinemic-euglycemic clamp, as previously described [22]. Pre-study conditions (overnight fasting, insulin lispro use, physical activity, etc.) were standardized to minimize variability. Female participants scheduled study visits during the follicular phase of the menstrual cycle (days 2–10) to minimize sex-hormone variability. In the parent trial, 17β-basal estradiol levels were similar between interventions and comparable to male levels, as previously reported [22].

### Insulin management and glycemic control

Participants continued their usual hybrid closed-loop insulin delivery during both dietary interventions. Basal rates, insulin-to-carbohydrate ratios, and correction factors remained stable. The goal was to achieve comparable mean continuous glucose monitor levels. Total daily insulin doses (TDD) were recorded from pump downloads to quantify differences in insulin exposure between interventions.

### Inpatient visit day procedures

Each dietary intervention concluded with a one-day inpatient visit at the Vanderbilt Clinical Research Center. On the day preceding each visit, participants consumed study-provided, pre-portioned standardized 2,000 kcal/day diets that matched the assigned macronutrient composition. They completed dinner between 5 and 7 PM and fasted overnight. Automated insulin delivery systems were maintained overnight with a target glucose of 110 mg/dL (6.1 mmol/L).

### Endothelial function assessment

Upon arrival (7:00 AM), vital signs and anthropometric data were obtained. Participants rested supine for 10 min in a quiet, temperature-controlled room before undergoing endothelial function assessment by brachial artery FMD. Baseline brachial artery diameter (D_base_) was measured using a Philips EPIQ 7 C ultrasound with an L12-3 transducer (Bothell, Washington, USA). A sphygmomanometer cuff was inflated to 200 mmHg to induce limb ischemia for 5 min, followed by ultrasound imaging of the brachial artery during reactive hyperemia for 10 s, starting 60 s after cuff deflation. After a 10-minute recovery, participants received 400 µg sublingual nitroglycerin to assess endothelium-independent dilation unless systolic blood pressure was < 100 mmHg. Vessel diameters at rest (D_base_) and peak hyperemia (D_peak_) were analyzed using Brachial Analyzer software (Medical Imaging Applications, Coralville, Iowa, USA). FMD images were analyzed by personnel blinded to diet assignments.

### Hyperinsulinemic-euglycemic clamp

Following vascular testing, participants underwent a 150-minute hyperinsulinemic-euglycemic clamp to assess insulin sensitivity. Venous catheters were placed in both arms, with one catheter site warmed to arterialize venous blood. Insulin lispro was infused at 40 mU/m²/min continuously for 150 min. Plasma glucose concentrations were measured every 5–10 min (YSI 2300, Yellow Springs Instruments), and a variable-rate 20% dextrose infusion was adjusted to maintain glucose at ≈ 95 mg/dL. IGFBP-1, IGFBP-2, IGFBP-3, total IGF-1, and free IGF-1 levels were measured from plasma samples collected twice thirty minutes apart at baseline, immediately before initiating the hyperinsulinemic, euglycemic clamp. We measured the same levels twice again under insulin-stimulated conditions during the final thirty minutes of the clamp.

### Analytical procedures

Arterialized venous blood samples were collected into tubes with potassium EDTA, chilled on ice, centrifuged (15 min at 3,000 RPM, 4 °C) and plasma was stored at -80 °C. Plasma insulin was measured by RIA (MilliporeSigma). IGFBP-1, IGFBP-2, IGFBP-3, total IGF-1, and free IGF-1 levels were measured by ELISA. Plasma samples were thawed, diluted, and analyzed in duplicate. Optical density (OD) values were measured at 450 nm using a Synergy H4 microplate reader (BioTek, USA) to quantify assay results. Additionally, IGFBP-1, IGFBP-2, and IGFBP-3 concentrations were analyzed for each participant in duplicate across both diets using the RayBiotech ELISA analysis template. Total IGF-1 and free IGF-1 levels were measured by Ansh Labs (Webster, TX, USA) using validated immunoassay (Cat# AL-122 for free IGF-1 and AL-121 for total IGF-1). Free IGF-1 levels were measured using a two-site antibody method to detect unbound IGF-1, while total IGF-1 was measured via a one-step sandwich immunoassay. Cytokine levels were measured using Luminex-based Milliplex Multiplex Panels (MilliporeSigma).

### Calculations

FMD, a measure of endothelium-dependent vascular function, served as an exploratory outcome. Because our previously published findings demonstrated no difference in FMD between dietary interventions [22], here we examined FMD primarily to explore potential relationships with changes observed in IGF axis variables.

Percent FMD was calculated using the following formula:

$$\:FMD\left(\mathrm{\%}\right)=\frac{{D}_{peak}-{D}_{base}}{{D}_{base}}$$

To quantify basal and insulin-stimulated IGFBP-1, IGFBP-2, IGFBP-3, total IGF-1, and free IGF-1 levels for each individual in each study, we calculated the mean of the two samples measured at baseline and the two samples measured at the end of the clamp, respectively.

### Statistical analysis

This study’s sample size was determined for the parent crossover trial’s primary endpoint (insulin sensitivity by clamp GIR) and key vascular outcome (FMD), as previously reported [22]. The present analyses of IGF axis measures were secondary analyses and were not independently powered. This report therefore emphasizes effect sizes and confidence intervals to describe the magnitude and precision of observed hormonal differences.

We utilized the Wilcoxon signed-rank test to compare IGFBP-1, IGFBP-2, IGFBP-3, total IGF-1, free IGF-1, and FMD across diets (GraphPad Prism, version 10.3.1). In an exploratory analysis, we used Spearman’s correlation to measure the strength of association between FMD and each IGF-1 level and binding protein in basal and insulin-stimulated states for each diet. Data are summarized as medians [25th -75th percentiles] unless otherwise specified.

## Results

### Participant characteristics

Fourteen adults with T1DM initially enrolled and completed the screening procedures. Two participants did not proceed beyond the run-in period because of scheduling and difficulty consistently tracking nutrient intake. We analyzed the remaining 12 individuals who completed both dietary phases. Baseline clinical characteristics are summarized in Table 1.

**Table 1:** Baseline characteristics of participants. Continuous variables are summarized as medians (25th-75th percentiles). Categorical and ordinal variables are expressed as percentages and counts

Baseline Characteristic	Participants (*n* = 12)
Male sex, % (n)	42% (5)
Age, years (25th-75th percentiles, total range)	33.9 (25.0–39.0, 23.3–49.9)
Weight, kg	75.3 (66.8–92.0)
Height, m	1.75 (1.68–1.88)
BMI, kg/m^2^	26.5 (21.7–28.4)
Waist-to-hip ratio	0.83 (0.75–0.88)
HbA1c, %	7.1 (6.6–7.7)
HbA1c, mmol/mol	54 (49–61)
Type 1 diabetes duration, years	20.5 (15.3–25.0)
Race, % (n)	
White	92% (11)
Black	8% (1)
Systolic blood pressure (mmHg)	120 (118–136)
Diastolic blood pressure (mmHg)	82 (78–90)
Heart rate (bpm)	65 (60–68)
Insulin analog used, % (n)	
Lispro	50% (6)
Aspart	42% (5)
Fast-acting insulin aspart	8% (1)

### Food consumption

**Fig. 1:**
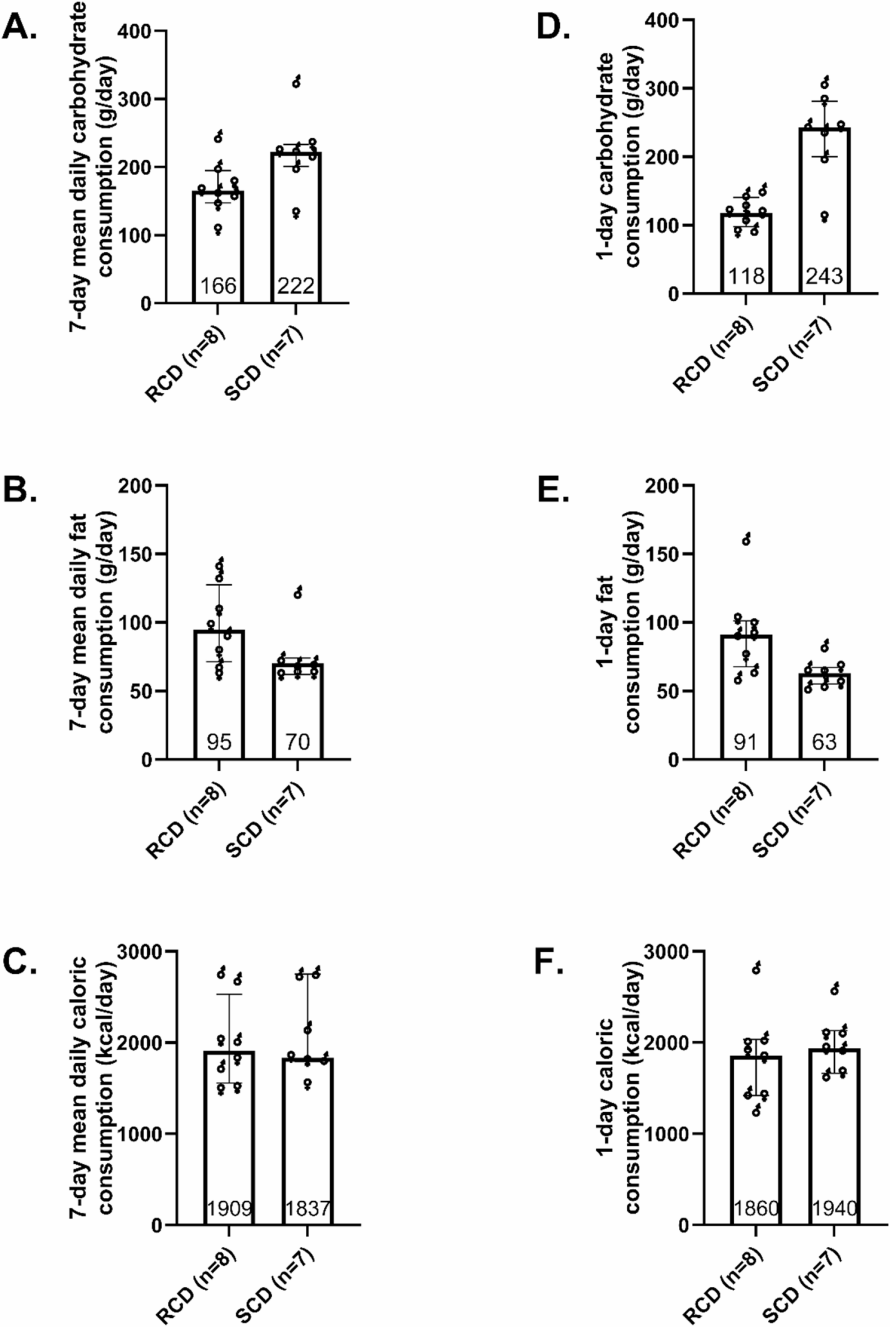
Dietary intake during interventions. Carbohydrate, fat, and caloric consumption during the one-week reduced-carbohydrate diet (RCD) and standard-carbohydrate diet (SCD) interventions preceding cardiometabolic experiments. The left column shows the average daily intake of carbohydrates (**A**), fats (**B**), and calories (**C**) during the one-week intervention. The right column shows carbohydrate, fat, and caloric intake during the 24 h immediately preceding experiments (**D**-**F**). Column scatter plots depict medians and 25th-75th percentiles

Throughout the seven-day RCD intervention, carbohydrate consumption was 25% lower and fat intake was 35% more than during the SCD (Fig. 1A-C). Dietary intake for the free-living days was derived from MyFitnessPal logs; eight participants completed logs for all six free-living days during the RCD period, and seven participants did so during the SCD period. During the 24-hours preceding each experiment, participants consumed study-provided standardized diets matched to the assigned macronutrient condition, and carbohydrate intake was 51% lower and fat intake was 44% higher with the RCD (Fig. 1D-F). Consistent with carbohydrate restriction, fasting β-hydroxybutyrate levels were higher after the RCD (260 µmol/L [137–282]) than after the SCD (123 µmol/L [79–205], median difference 54 µmol/L, 95% CI 2.7–147 µmol/L).

### Glycemic control and insulin delivery

During the week preceding each study visit, participants required 16% less total daily insulin while on the RCD compared to the SCD. Further, during the 24 h immediately preceding each experiment, when participants consumed study-supplied standardized meals, insulin requirements fell 24% on the RCD compared with the SCD. Despite the difference in insulin dosing, continuous glucose monitoring data showed similar mean glucose levels between the two dietary interventions (160 mg/dL [145–165] for RCD vs. 150 mg/dL [135–164] for SCD, median difference [RCD-SCD] 5 mg/dL, 95% CI -5 to 19 mg/dL, *p* = 0.14).

### Insulin, glucose, and inflammatory marker concentrations during experiments

Fasting plasma insulin concentrations were similar between interventions (56 pmol/L [40–74] after RCD vs. 63 pmol/L [43–83] after SCD). During the hyperinsulinemic phase of the clamp, insulin levels rose approximately fivefold and were comparable between interventions (317 pmol/L [279–371] for RCD vs. 330 pmol/L [218–396] for SCD). Fasting plasma glucose concentrations were also similar (124 mg/dL [113–131] after RCD vs. 115 mg/dL [101–140] after SCD), and clamp glucose was maintained near target in both studies (92 mg/dL [90–95] with RCD vs. 96 mg/dL [91–100] with SCD). Baseline inflammatory cytokines and endothelial activation markers (including IL‑6, TNFα, soluble adhesion molecules, and PAI‑1) were similar between interventions (Supplemental Table 2). Detailed clamp outcomes and additional metabolic and hormonal measurements were reported previously [22].

### IGFBP-1, IGFBP-2 and IGFBP-3 levels

#### IGFBP-1 was higher following RCD during basal and insulin-stimulated conditions

**Fig. 2:**
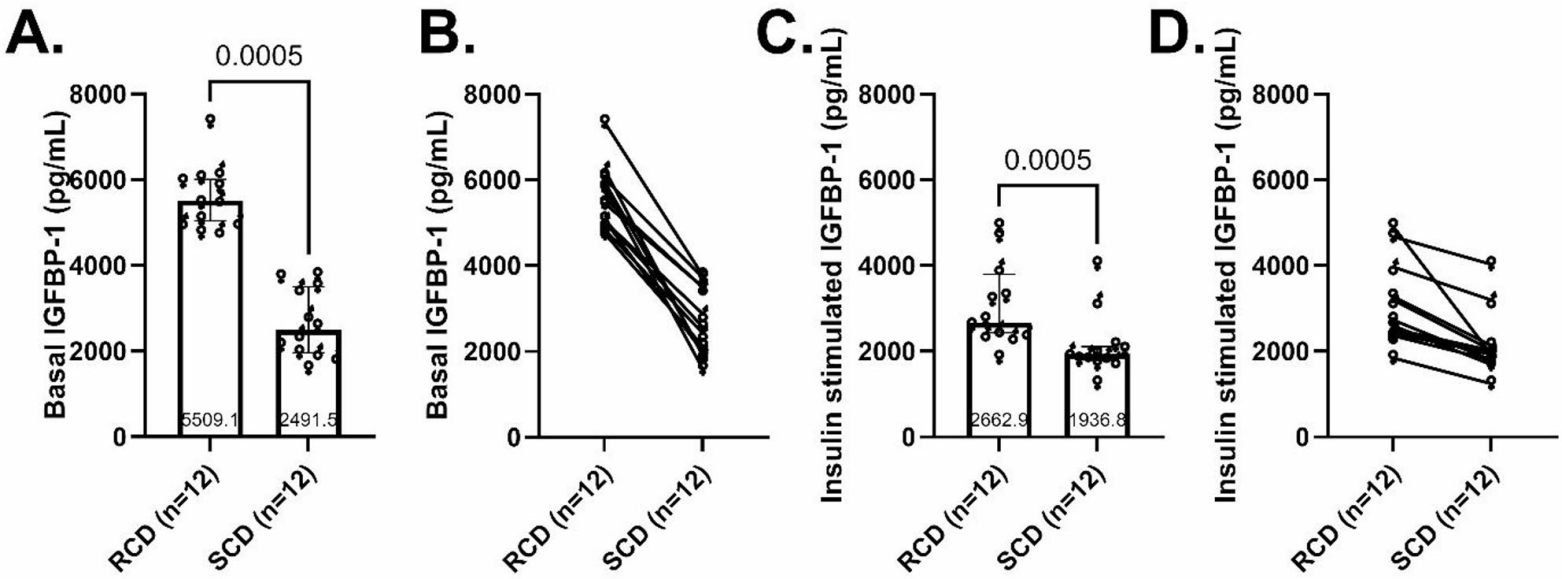
IGFBP-1 levels. Plasma concentrations of IGFBP-1 during basal (**A**-**B**) and insulin-stimulated conditions (**C**-**D**) following one-week reduced carbohydrate diet (RCD) and standard carbohydrate diet (SCD) interventions. Column scatter plots (**A** and **C**) show medians, 25th-75th percentiles, and p-values. Paired dot plots (**B** and **D**) depict within-participant changes

Baseline plasma IGFBP-1 concentrations were significantly higher on the RCD compared to the SCD (5,509 pg/mL [5,043 − 6,010] vs. 2,492 pg/mL [1,964-3,501], respectively, median difference 2,570 pg/mL, 95% CI 2,161-3,993, *p* < 0.0005, Fig. 2A) and were higher in every participant (Fig. 2B). The hyperinsulinemic conditions of the clamp suppressed IGFBP-1 levels and attenuated the difference between interventions (2,663 pg/mL [2,436-3,797] for RCD vs. 1,937 pg/mL [1,797-2,109] for SCD, median difference 709 pg/mL, 95% CI 545-1,144, *p* < 0.0005, Fig. 2C-D).

#### IGFBP-2 was modestly higher following RCD during basal and insulin-stimulated conditions

**Fig. 3:**
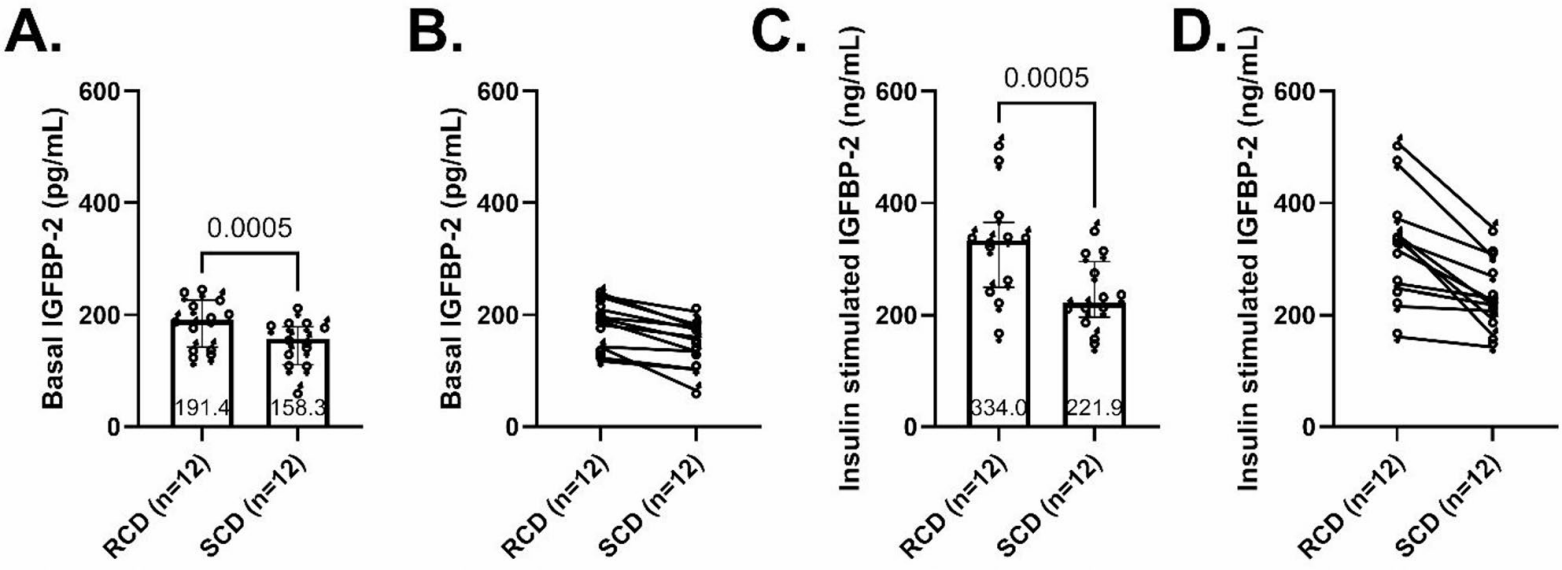
IGFBP-2 levels. Plasma concentrations of IGFBP-2 during basal (**A**-**B**) and insulin-stimulated conditions (**C**-**D**) following one-week reduced carbohydrate diet (RCD) and standard carbohydrate diet (SCD) interventions. Column scatter plots (**A** and **C**) show medians, 25th-75th percentiles, and p-values. Paired dot plots (**B** and **D**) depict within-participant changes

Differences in basal IGFBP-2 concentrations between interventions were smaller than differences seen with IGFBP-1 but were statistically significant. Median basal IGFBP-2 was 191.4 pg/mL [142.7-226.7] following RCD and was 158.3 pg/mL following SCD [111.2-179.4] (median difference 31.5 pg/mL, 95% CI 16.2–54.2, *p* < 0.0005, Fig. 3A). As with IGFBP-1, basal IGFBP-2 levels for every participant were higher following the RCD compared with the SCD (Fig. 3B). During the hyperinsulinemic clamp, IGFBP‑2 was 334.0 pg/mL [249.5-365.1] after RCD vs. 221.9 pg/mL [196.4-295.1] after SCD (median difference 77.3, 95% CI 25.3-152.2, *p* < 0.0005, Fig. 3C–D).

#### IGFBP-3 was lower following RCD during basal and insulin-stimulated conditions

**Fig. 4:**
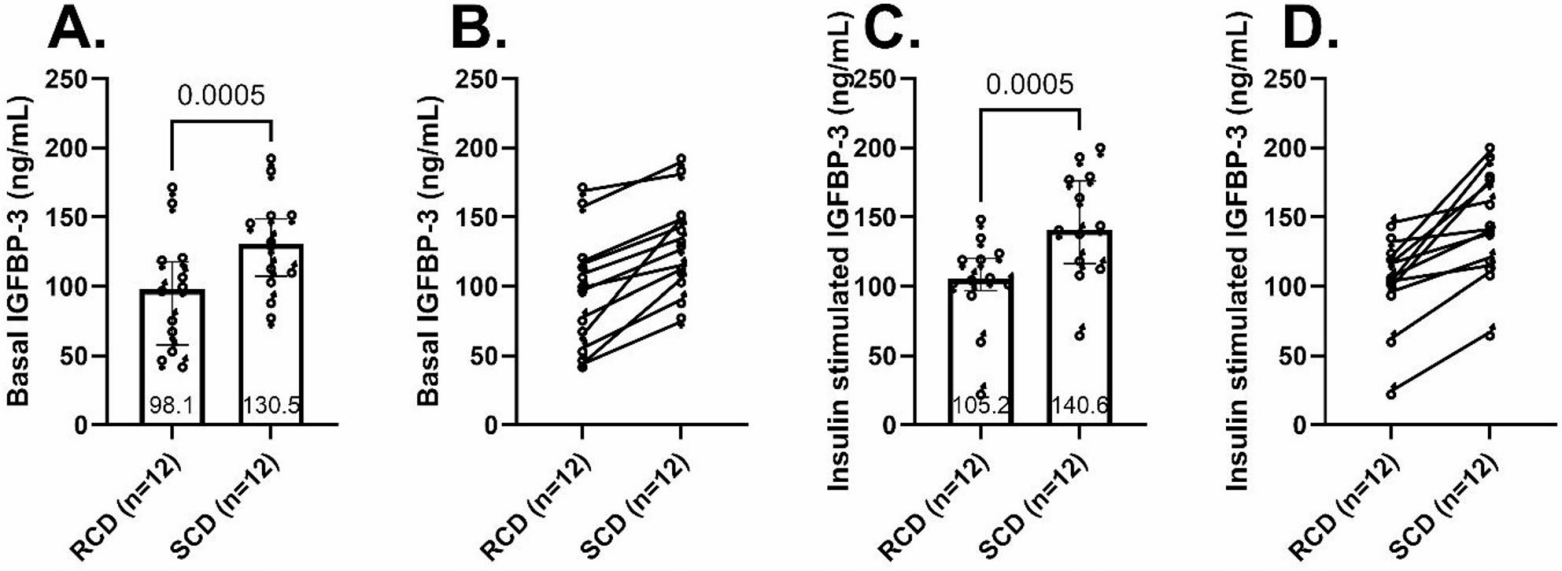
IGFBP-3 levels. Plasma concentrations of IGFBP-3 during basal (**A**-**B**) and insulin-stimulated conditions (**C**-**D**) following one-week reduced carbohydrate diet (RCD) and standard carbohydrate diet (SCD) interventions. Column scatter plots (**A** and **C**) show medians, 25th-75th percentiles, and p-values. Paired dot plots (**B** and **D**) depict within-participant changes

Basal IGFBP-3 levels were lower with the RCD compared to the SCD (98.1 ng/mL [57.9-117.5] vs. 130.5 ng/mL [107.1-148.7], respectively, median difference − 30.6 ng/mL, 95% CI: -35.1 to -25.6, *p* < 0.0005, Fig. 4A). IGFBP-3 was lower in every participant following the RCD compared with the SCD (Fig. 4B). Median IGFBP-3 concentrations rose minimally during the hyperinsulinemic, euglycemic clamp and levels remained lower with RCD compared with SCD (Fig. 4C-D). Under the insulin stimulated conditions, IGFBP-3 was 105.2 ng/mL [96.9-120.1] after RCD vs. 140.6 ng/mL [116.4-176.2] after SCD (median difference − 37.9, 95% CI -76.4 to -15.6, *p* < 0.0005).

### Total IGF-1 was lower following RCD, and free IGF-1 levels were more variable and did not differ between interventions

**Fig. 5:**
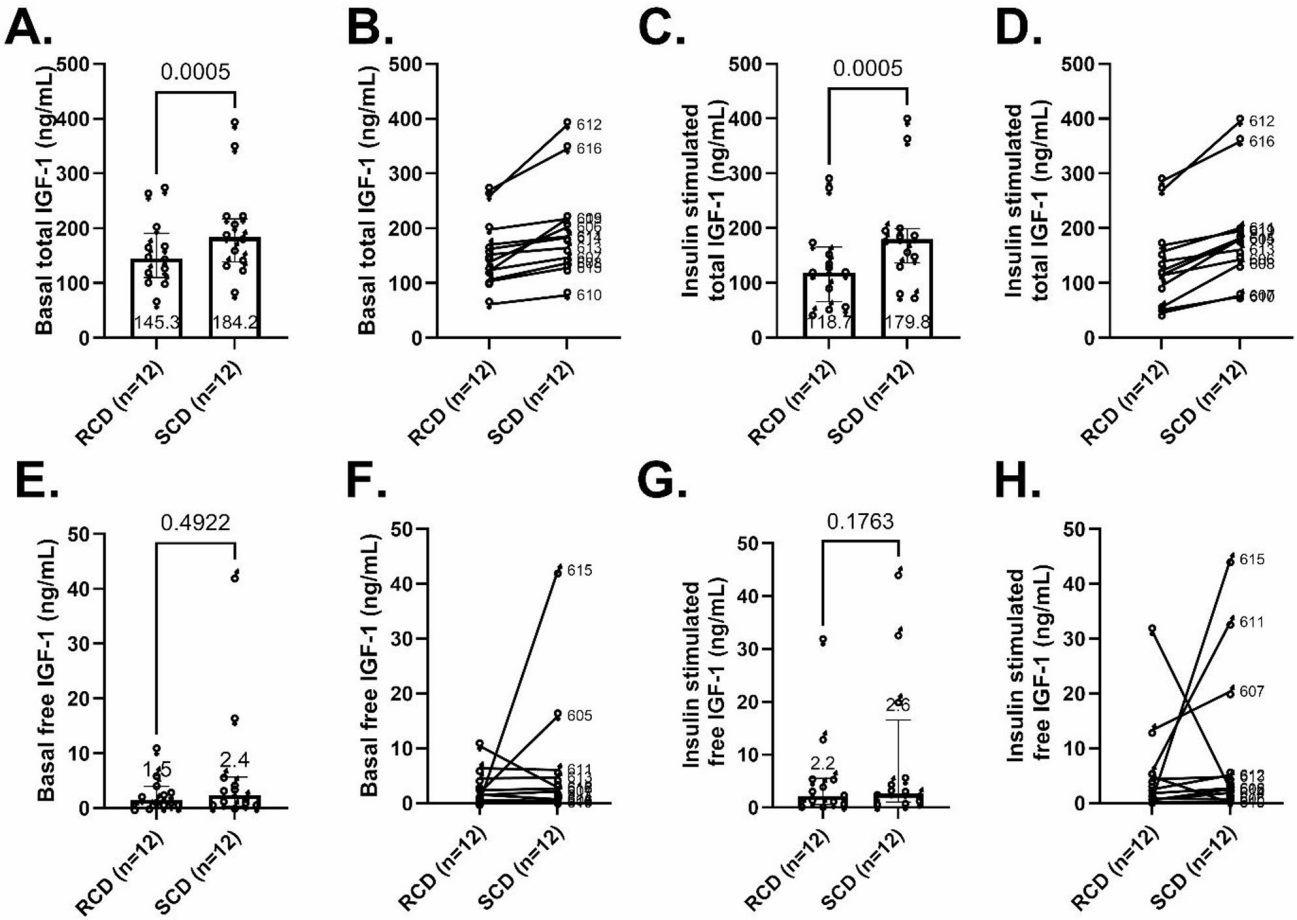
Total and free IGF-1 levels. Plasma concentrations of total (**A**-**D**) and free (**E**-**H**) IGF1 during basal (**A**-**B**, **E**-**F**) and insulin-stimulated conditions (**C**-**D**, **G**-**H**) following one-week reduced carbohydrate diet (RCD) and standard carbohydrate diet (SCD) interventions. Column scatter plots (**A**, **C**, **E**, and **G**) show medians, 25th-75th percentiles, and p-values. Paired dot plots (**B**, **D**, **F**, and **H**) depict within-participant changes

Total IGF-1 levels were lower following the RCD compared to the SCD at baseline (145.3 ng/mL [110.0-191.0] vs. 184.2 ng/mL [139.0-217.4], median difference − 23.4 ng/mL, 95% CI: -76.6 to -16.4, *p* < 0.0005, Fig. 5A-B). During the hyperinsulinemic clamp, the total IGF-1 decreased modestly for RCD and changed minimally for SCD, with levels remaining lower with RCD than SCD (118.7 ng/mL [65.7-165.7] after RCD vs. 179.8 ng/mL [136.4-198.9] after SCD, median difference − 51.2, 95% CI -78.1 to -26.2, *p* < 0.0005, Fig. 5C-D). In exploratory analyses, the between-diet difference in insulin dose during the final 24 h correlated with the between-diet difference in fasting total IGF‑1 (ρ = 0.70, *p* = 0.025), whereas no significant associations were observed for ΔIGFBP‑1, ΔIGFBP‑2, or ΔIGFBP‑3 (all *p* > 0.2).

Median baseline and insulin-stimulated free IGF-1 levels were more variable and showed no statistically significant differences between interventions (Fig. 5E-H). In the basal state, free IGF-1 was 1.5 ng/mL [0.2-4.0] after RCD vs. 2.4 [0.4–5.7] after SCD (median difference − 0.05, 95% CI -0.3-0.7, *p* = 0.49). Under insulin-stimulated conditions, free IGF-1 was 2.2 ng/mL [0.6–5.5] after RCD vs. 2.6 [1.0-16.6] after SCD (median difference − 1.1, 95% CI -0.1-7.0, *p* = 0.18).

Because sex hormones could influence GH-IGF physiology, we performed an exploratory sex-stratified summary of basal IGF axis parameters. The direction of intervention-associated changes was similar between men and women (Supplemental Table 3).

### Flow-mediated dilation was similar between RCD and SCD

**Fig. 6:**
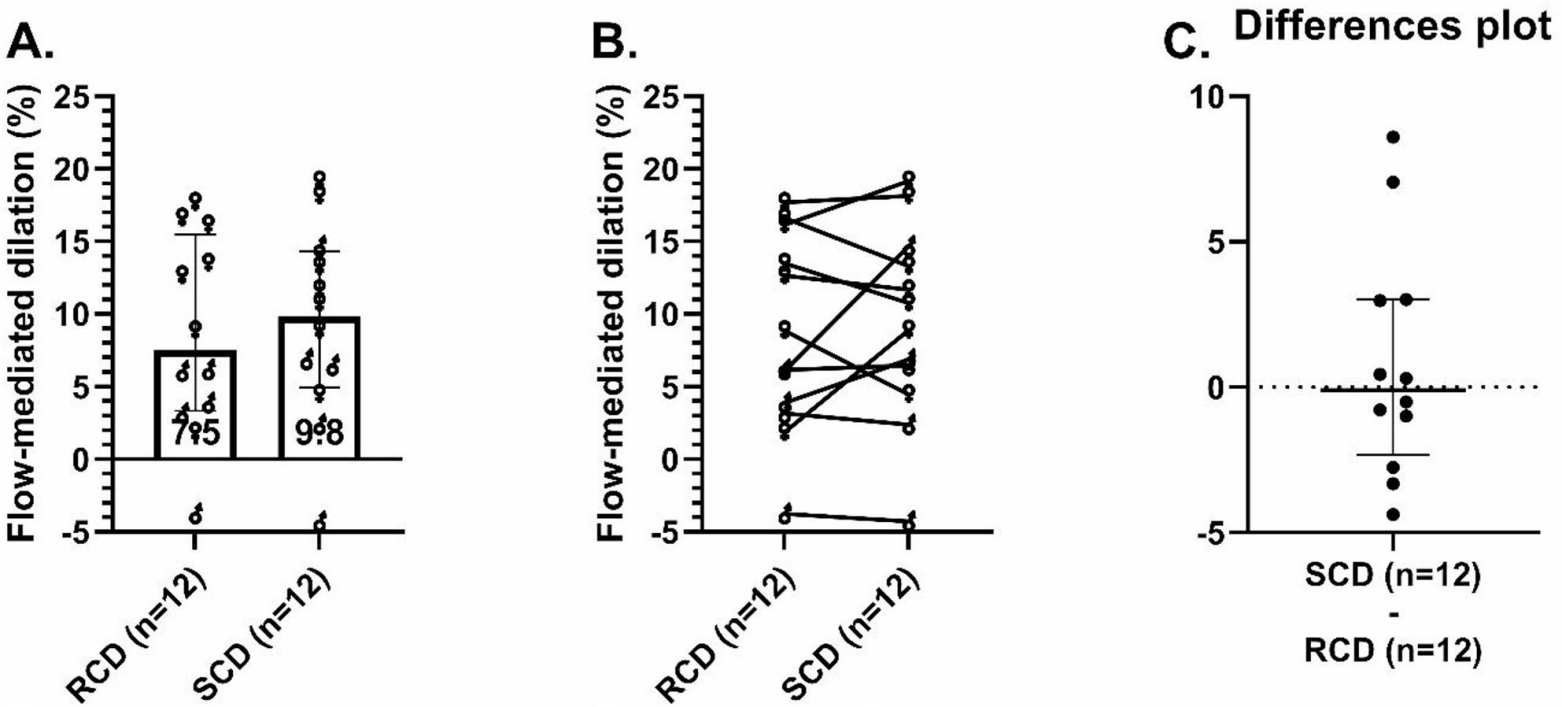
Endothelial function following interventions. Endothelium-dependent flow-mediated dilation (FMD) after the reduced-carbohydrate diet (RCD) and standard-carbohydrate diet (SCD) interventions. Plot **A** shows individual FMD data, intervention medians, and the 25th-75th percentiles. Plot **B** shows within-participant changes in FMD between interventions. Plot **C** shows within-participant differences in FMD between interventions (SCD minus RCD) for each individual, along with median and 25th-75th percentiles for these differences

FMD did not significantly differ between interventions (RCD: 7.50% [3.25–15.5%], SCD: 9.81% [4.96–14.3%]; *p* = 0.91; median difference = 0.10%, 95% CI − 2.76 to 3.01; Fig. 6A–C). FMD did not correlate with basal or insulin‑stimulated levels of IGFBP‑1, IGFBP‑2, IGFBP‑3, total IGF‑1, or free IGF‑1 after either diet.

## Discussion

In this randomized, crossover trial of 12 adults with T1DM, one week of an RCD lowered the total daily insulin dose and shifted the IGF axis. Total IGF-1 and IGFBP-3 decreased, while IGFBP-1 and IGFBP-2 increased compared with the SCD. Notably, these changes occurred without clinically meaningful differences in glycemia, insulin sensitivity, or endothelial function.

The IGF axis pattern on the RCD, where total IGF-1 and IGFBP-3 decreased and IGFBP-1 increased (and IGFBP-2, modestly), is consistent with reduced hepatic insulin exposure. Portal insulin is permissive for GH-stimulated hepatic IGF-1 production. When portal insulin decreases, the liver becomes relatively GH-resistant, IGF-1 output falls, and hepatic IGFBP-1 secretion rises. Hepatic-vein catheterization studies show that circulating IGFBP-1 is liver-derived and acutely suppressible by insulin, supporting its use as a sensor of hepatic insulin exposure [21]. In T1DM, increasing hepatic insulin exposure via intraportal or intraperitoneal insulin delivery raises IGF-1 and IGFBP-3 and lowers IGFBP-1 [9–11]. Thus, by lowering the total insulin dose, the RCD likely further reduced portal insulin exposure, producing coordinated changes across the IGF axis in the direction expected with hepatic underinsulinization. Consistent with this framework, participants who reduced insulin more in the final 24 h also showed a larger decrease in fasting total IGF‑1.

Despite clear within-participant shifts in the IGF axis, we did not detect corresponding differences in conduit artery FMD. We expected that lowering peripheral insulin exposure would improve endothelial function, because peripheral hyperinsulinemia during euglycemic clamps reduces FMD in humans [30, 31]. We reasoned this improvement could plausibly occur through reduced nitric oxide bioactivity and increased endothelin-1/oxidative stress signaling. In prior hyperinsulinemic clamp studies, endothelial dysfunction emerged during sustained insulin exposure over hours, and antioxidant rescue supports a role for oxidative stress [30, 31]. However, in our study, endothelial function was measured after an overnight fast under standardized conditions using automated insulin delivery, and fasting insulin exposure on the morning of testing was similar between interventions. In addition, the RCD shifted IGF-1 axis biomarkers in a direction that could counterbalance any favorable effect of reduced daytime insulin exposure on endothelial function. Mechanistically, IGF‑1 can act directly on vascular endothelium via IGF‑1 receptors to promote nitric oxide bioavailability and endothelial nitric oxide synthase signaling. Endothelial cells also express insulin receptors and IGF‑1 receptors can form hybrid receptors, creating overlap and cross talk between insulin- and IGF‑1-mediated signaling [32, 33]. This signaling overlap, together with binding-protein buffering of free IGF‑1, may help explain why the observed diet-associated shift in total IGF‑1/IGFBPs did not translate into a detectable change in conduit-artery FMD over one week. Finally, our small sample size limited power to detect modest endothelial effects.

Clinically, these findings are relevant because they show that a modest, achievable reduction in insulin requirements, achieved without meaningful differences in glycemia, can shift IGF axis biomarkers within one week in adults with T1DM. The coordinated direction of change—lower total IGF-1 and IGFBP-3 with higher IGFBP-1 and IGFBP-2—matches the IGF axis signature attributed to relative hepatic underinsulinization in T1DM. This direction is opposite to the direction reported when portal insulin exposure is increased by intraportal or intraperitoneal insulin delivery [2, 3, 9–11]. This observation has potential clinical importance because IGF-1 supports vascular homeostasis through effects on endothelial signaling, oxidative stress, and inflammation [8, 12]. Moreover, IGFBP-1 show context-dependent associations with cardiovascular outcomes in observational cohorts [3, 15]. Importantly, our data do not establish short-term vascular benefit or harm: FMD was an exploratory surrogate, and we did not detect a difference in FMD between interventions over one week. Rather, our results identify the IGF axis as a sensitive physiologic readout of hepatic insulin exposure that future, longer-duration studies should incorporate when evaluating vascular endpoints and clinical outcomes.

Strengths of the study include a randomized crossover design with each participant serving as his or her own control and nearly matched glycemia between interventions, isolating effects linked to insulin exposure rather than glucose. Standardized meals before testing, blinded vascular measurements, and paired clamp sampling added methodological rigor.

Limitations include small sample size, single-center recruitment, and one-week interventions that preclude long-term inference. Because standardized meals were provided in the final 24 h and IGFBP‑1 is acutely insulin-responsive, we cannot distinguish acute pre-visit effects from cumulative week-long effects. In addition, portal insulin exposure was inferred rather than measured, we did not assess GH pulsatility, and variability in free IGF-1 assays that may have obscured small between-diet differences. Finally, because the RCD simultaneously changed macronutrient composition and reduced exogenous insulin dosing, our crossover design cannot disentangle direct macronutrient effects from insulin-mediated effects on the IGF axis. Given the established insulin regulation of hepatic IGFBP‑1 and IGF‑1 production, and the concordant direction of change seen in portal-delivery studies, we interpret reduced hepatic insulin exposure as the most parsimonious explanation, while acknowledging possible diet-composition contributions.

Future studies should determine whether habitual carbohydrate restriction in T1DM durably modifies the IGF axis and whether those changes affect vascular biology and clinical outcomes. We advocate for longer interventions with standardized diets and adequately powered trials embedding cardiovascular endpoints or validated surrogates.

## Conclusions

In adults with T1DM, short‑term carbohydrate reduction lowered insulin dose and shifted the IGF axis toward a pattern consistent with reduced portal insulinization, without detectable changes in endothelial function. These findings support the IGF axis as a sensitive physiologic readout of hepatic insulin exposure under similar glycemia and motivate longer, mechanistically anchored studies.

## Supplementary Information

Below is the link to the electronic supplementary material.

Supplementary Material 1

## Data Availability

Data are provided within the manuscript. The datasets generated or analyzed during this study are available from the corresponding author upon reasonable request. The full trial protocol is also available upon reasonable request. No applicable resources were generated.

## Abbreviations

CGM: Continuous glucose monitor
CI: Confidence interval
EDTA: Ethylenediaminetetraacetic acid
ELISA: Enzyme‑linked immunosorbent assay
FMD: Flow‑mediated dilation
GH: Growth hormone
IGF‑1: Insulin‑like growth factor‑1
IGFBP‑1/‑2/‑3: Insulin‑like growth factor‑binding protein‑1/‑2/‑3
OD: Optical density
RCD: Reduced‑carbohydrate diet
RIA: Radioimmunoassay
SCD: Standard‑carbohydrate diet
TDD_insulin_: Total daily dose of insulin
