# Supplementary material for: A one-week reduced-carbohydrate diet lowers insulin requirements and shifts the IGF axis with no detectable short-term change in endothelial function in a randomized, crossover trial of adults with type 1 diabetes
Source: Cardiovasc Diabetol Endocrinol Rep. 2026 Mar 26;12:13. doi: 10.1186/s40842-026-00276-6 (PMC13020340; doi:10.1186/s40842-026-00276-6)
Supplement: Supplementary file 1 — Supplementary Material 1 [file 40842_2026_276_MOESM1_ESM.docx]

# SUPPLEMENTAL MATERIAL

1. Supplemental Tables (p. 2-4)

# SUPPLEMENTAL TABLES:


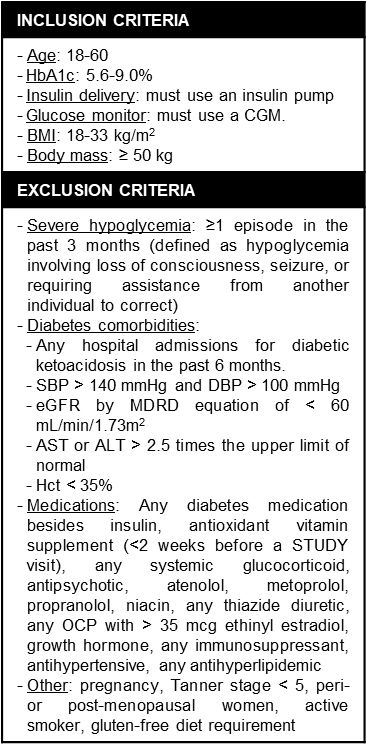


Supplemental Table 1. Inclusion and exclusion criteria. SBP = systolic blood pressure. DBP = diastolic blood pressure. eGFR = estimated glomerular filtration rate. MDRD = modification of diet in renal disease. AST = aspartate aminotransferase. ALT = alanine aminotransferase. Hct = hematocrit. OCP = oral contraceptive pill.

|  | RCD (n=12) | SCD (n=12) | Median paired difference (RCD – SCD) |
| --- | --- | --- | --- |
| IL-1α (pg/mL) | 5.9  [1.3-40] | 8.4  [2.4-27] | -1.9 |
| IL-1β (pg/mL) | 5.6  [2.2-9.2] | 3.4  [1.4-7.4] | 0 |
| IL-6 (pg/mL) | 1.2  [0.4-2.8] | 2.0  [0.7-4.0] | -0.5 |
| TNF-α (pg/mL) | 9.5  [5.8-16] | 11  [7.4-19] | -2.2 |
| VEGF-A (pg/mL) | 16.5  [6.74-51.7] | 26.4  [10.1-49.2] | -6.65 |
| sICAM (ng/mL) | 113  [93.1-148] | 130  [97.1-160] | -9.16 |
| sVCAM (ng/mL) | 772  [697-785] | 723  [697-795] | 20.0 |
| Fibrinogen (g/mL) | 20.9  [19.4-22.4] | 24.0  [19.3-27.6] | -3.47 |
| PAI-1 (ng/mL) | 11.0  [5.01-20.9] | 17.0  [7.24-23.6] | -4.72 |

Supplemental Table 2. Basal plasma concentration of inflammatory mediators. Data are summarized as medians [25th-75th percentiles]. RCD = reduced carbohydrate diet, SCD = standard carbohydrate diet, IL = interleukin, TNF = tumor necrosis factor, VEGF = vascular endothelial growth factor, sICAM = soluble intercellular adhesion molecule, sVCAM = soluble vascular cell adhesion molecule, PAI = plasminogen activator inhibitor

|  |  | Men (n=5) |  |  | Women (n=7) |  |
| --- | --- | --- | --- | --- | --- | --- |
|  | **RCD** | **SCD** | **Median paired difference (RCD – SCD)** | **RCD** | **SCD** | **Median paired difference (RCD – SCD)** |
| IGFBP-1, pg/mL | 5571  [4943-6097] | 2421  [1928-3187] | 2425 | 5447  [5052-6031] | 2562  [1990-3722] | 2309 |
| IGFBP-2, pg/mL | 193.6  [162.6-235.9] | 160.6  [110.5-181.3] | 48.9 | 189.2  [123.3-209.7] | 135.3  [103.3-179.5] | 14.9 |
| IGFBP-3, ng/mL | 77.9  [49.9-112.5] | 112.2  [98.0-138.6] | -34.4 | 99.1  [64.8-157.4] | 148.5  [115.2-180.9] | -30.6 |
| total IGF-1, ng/mL | 123.8  [104.7-166.2] | 146.4  [132.0-184.2] | -22.6 | 152.1  [121.7-259.1] | 217.1  [164.2-345.0] | -63.8 |
| free IGF-1, ng/mL | 1.5  [0.2-4.0] | 2.2  [0.5-24.2] | -0.1 | 1.9  [0.1-4.5] | 2.7  [0.1-4.6] | 0.0 |

Supplemental Table 3. Sex-stratified summary of plasma basal IGF axis concentrations. Data are summarized as medians [25th-75th percentiles]. RCD = reduced carbohydrate diet, SCD = standard carbohydrate diet, IGF = insulin-like growth factor, IGFBP = insulin-like growth factor binding protein
